# Underlining neighbourhood perception: a possible risk factor for dementia that deserves more attention

**DOI:** 10.1093/braincomms/fcae037

**Published:** 2024-03-12

**Authors:** Barış Sevi, Ángela Gutiérrez, Graciela Muniz-Terrera

**Affiliations:** Heritage College of Osteopathic Medicine, Ohio University, Athens, OH 45701, USA; Department of Psychology, MEF University, 34396 Istanbul, Turkey; Heritage College of Osteopathic Medicine, Ohio University, Athens, OH 45701, USA; Heritage College of Osteopathic Medicine, Ohio University, Athens, OH 45701, USA; Edinburgh Dementia Prevention, University of Edinburgh, Edinburgh EH8 9YL, UK

## Abstract

This essay highlights the interplay between the neighbourhood structural environment and neighbourhood perceptions on dementia by articulating how an individual’s perception of neighbourhood, with respect to their individual differences, may provide key insights to understand the link between the neighbourhood and dementia.

More than 50 million people are currently living with dementia, and it is expected that this number will exceed 150 million by 2050.^1^ In addition to cognitive impairment, dementia has physiological, economic and social impacts, and these impacts are not only on the individual but also on their social network (e.g. families and friends). For a syndrome that has such multifaceted consequences and because of the exponentially increasing number of people it affects, understanding the antecedents and identifying opportunities to delay or prevent it are essential.

Although genetic risk factors have been identified for dementia, several modifiable risk factors have also been shown to be associated with dementia.^1^ A broader understanding of the aetiology of non-genetic risk factors, the ‘exposome’, would be beneficial in identifying individuals at increased risk of dementia and in informing the design of effective interventions to delay or prevent the onset of disease. In this essay, we underline a specific external factor of the exposome: neighbourhood perception. Specifically, we highlight the interplay between the neighbourhood structural environment and neighbourhood perceptions of dementia by articulating how an individual’s perception of neighbourhood, with respect to their individual differences, may provide key insights to understand the link between the neighbourhood and dementia.

The neighbourhood can influence social contact through its built/structural environment (e.g. green spaces) and its social environment (e.g. interaction with neighbours).^2^ Social contact is a modifiable risk factor that has been identified as protective against developing dementia as it can contribute to the build-up of more cognitive reserve, helping to tolerate more neuropathology.^1,3^ Yet, social contact is dependent on the social environment in which a person lives. In the study of neighbourhood, the structural environment has gained the most attention, yet research on the social aspects of neighbourhoods is emerging. Unlike neighbourhood structural characteristics, which can be assessed using data about green spaces, population density and other characteristics that are easily quantifiable, social characteristics can be harder to quantify. Indeed, the quantification of occurrences and quantities of social contact may not be sufficient to adequately capture the value and impact of social contact. The quality of social interactions has important implications for cognitive aging, with positive and strained relationships differentially affecting health and well-being.^4^ Therefore, an approach that considers the perception of the neighbourhood environment would help in disentangling how neighbourhood social environment affects individuals.

A measure of neighbourhood perception was developed and validated by Cagney *et al.*,^5^ building on the theories of collective efficacy and social disorganization. Collective efficacy emphasizes the ability of a community to come together and form mutual trust, while social disorganization builds on a criminological perspective and focuses on the influence of social and physical disorder. Accordingly, the measure has two major factors. ‘Neighbourhood social cohesion and change’ pertains more to the beneficial aspects (i.e. observations of and interactions with neighbours), and ‘social and physical disorder’ pertains more to the problematic aspects of the neighbourhood (i.e. neighbourhood problems and unsafe conditions). Neighbourhood perceptions have been shown to be associated with physical and psychological health. Further, even though the framework is relatively new, there have been studies that have shown some connection with measures of brain health. For example, high social cohesion has been associated with better global cognition, episodic memory and better cognitive performance.^6^ On the other side, higher physical disorder was associated with worse episodic memory.^7^ Underlining the importance of social connectedness as a protective factor, we subscribe to the concept that neighbourhood perception is a relevant factor in the study of dementia risk.

The mechanism explaining the association between neighbourhood perception and dementia risk is through its association with social contact, a modifiable risk factor, that can help in building cognitive reserve. Notably, this mechanism also differs across racial and ethnic minoritized groups. Some racial and ethnic minoritized groups are at increased risk for dementia, and there have been attempts to identify and address these inequalities.^1^ Projections show that racial and ethnic minoritized groups, especially Hispanics, African Americans and Asians, will constitute a higher portion of older individuals, making it even more important to understand how racial–ethnic disparities contribute to dementia risk.^8^ To aid in these inequalities, providing solutions for structural differences of neighbourhoods and making changes to differences that are more visible to the eye may be the first solution. However, what we would like to emphasize is that even if a neighbourhood’s structural inequalities are remedied, they may not achieve the desired effects.

Increasing the number of green spaces, lowering population density and creating a better transportation infrastructure are all sound and possibly good investments for a better neighbourhood and living for their inhabitants. However, the returns for these investments are not easy to calculate, as having a structural change does not always result in getting back the desired effects. There are many other factors at play, as suggested by ‘the diminishing returns hypothesis’^9^ that provides a good example of this phenomenon. In their study, Farmer and Ferraro^9^ compared the health outcomes of Black and White Americans who were all of high socioeconomic status (SES). Their findings showed that Black Americans did not receive the same health returns as White Americans.^9^ Findings underscore how similar socioeconomic context still yields health disparities for racial and ethnic minoritized groups, relative to their White counterparts. A structural focus on neighbourhoods may evaluate all areas with high SES as the same, but findings show that not all people with high SES get the same returns for their health. Neighbourhood structural factors are useful with accessible data sources like the census. However, neighbourhood structural factors can be considered as more rigid and may lead to missing out on differences for minoritized groups and individual-level variances. Using a framework that considers individuals’ perceptions allows for the consideration of their perception of their immediate environment and how that perception influences health. A perception-focused framework grants us to examine if the neighbourhood structural changes (if made) reflect on the people, which would be particularly essential for minoritized groups who might not get the desired returns for their health.

The current evidence about the connection of neighbourhood perception and dementia is scarce, and generalizability is limited due to possible culture-specific findings. Although dementia affects people all around the world, a one-size-fits-all prevention method may not be the best approach. For example, it is known that the income level of countries is associated with dementia, where individuals from lower income countries are at higher risk.^10^ In planning future research, considering the effects of the different factors within the exposome and using a tailored approach for specific populations and individuals may be more fit. In doing so, studies should consider covariates that are an individual’s certain attitudes and behaviours, as well as macro-level cultural values. For individual covariates in the connection between neighbourhood perception and dementia risk, considering the association neighbourhood perception has with other modifiable risk factors for dementia can be useful. Other than social contact, neighbourhood perception can be in association with the risk factors of depression and physical activity. Higher depression occurrence and lower levels of physical activity have been identified as modifiable risk factors.^1^ A neighbourhood with a perception of lower levels of cohesion, where there is a perception of less support, can be associated with more depressive thoughts. A neighbourhood perception with more disorder can be associated with less physical activity, where individuals are reluctant to go outside for physical exercises or walks. Although social contact may be the main factor associated with neighbourhood perception, considering other modifiable risk factors can improve dementia risk models. As for macro-level covariates, the culture of populations can be a major contributor. The spectrum of individualism–collectivism, especially, would be appropriate for social contact. Collectivistic populations have norms that facilitate more social contact within the community members, while individualism promotes individual independence. A lower perception of cohesion can be more of a risk factor in collectivistic cultures, while in individualistic cultures, perception of cohesion may not have as much value. To approach more cohesive models, including these covariates or third factors would be beneficial.

Building on the acquired knowledge, interventions can be designed focusing on neighbourhood perception that would help improve brain reserve and promote resilience for dementia. Interventions that focus on improving individuals’ understanding and perceptions of the ongoing development in their neighbourhood can be one of the ways to help individuals engage with the changes in their neighbourhood. For example, to promote the physical activity of residents, promoting an environment with less disorder may not be enough. Interventions and better information could be shared with local residents to improve their perception and understanding of the changes that have occurred in their neighbourhood so they can safely engage in exercise. Helping improve the perception of residents in the neighbourhood would help them have a better and possibly more accurate perception of their neighbourhood that can translate into lifestyle changes that increase cognitive reserve and reduce dementia risk.

As research progresses on dementia, the role of potentially modifiable factors is better understood, and it is now accepted that they have a more significant role than previously thought in preventing dementia. Even in the two recent reports of the ‘Lancet Commission’, an increase in the role of potentially modifiable factors is seen from 35 to 40%, where the role of social contact increased from 2 to 4%.^1,10^ To aid an impairment with no cure, exploring all possible paths that delay or prevent dementia, or for individuals with dementia, ways to have a better experience, should be the goal. Neighbourhoods are in the immediate environment of people. Here, we have briefly documented how the study of perceived cohesion and neighbourhood disorder can be a promising pathway to reduce dementia prevalence.
